# A Cell Cycle-Related 13-mRNA Signature to Predict Prognosis in Hepatocellular Carcinoma

**DOI:** 10.3389/fonc.2022.760190

**Published:** 2022-03-28

**Authors:** Yang Zhou, Dengliang Lei, Gangli Hu, Fang Luo

**Affiliations:** ^1^Department of Hepatobiliary Surgery, The First Affiliated Hospital of Chongqing Medical University, Chongqing, China; ^2^Central Laboratory, The First Affiliated Hospital of Chongqing Medical University, Chongqing, China

**Keywords:** cell cycle, hepatocellular carcinoma, mRNA signature, GSEA, prognosis, TICRR

## Abstract

We aimed to propose a cell cycle-related multi/mRNA signature (CCS) for prognosis prediction and uncover new tumor-driver genes for hepatocellular carcinoma (HCC). Cell cycle-related gene sets and HCC samples with mRNA-Seq data were retrieved from public sources. The genes differentially expressed in HCCs relative to normal peritumoral tissues were extracted through statistical analysis. The CCS was constructed by Cox regression analyses. Predictive capacity and clinical practicality of the signature were evaluated and validated. The expression of the function-unknown genes in the CCS was determined by RT-qPCR. Candidate gene *TICRR* was selected for subsequent validation through functional experiments. A cell cycle-related 13-mRNA signature was generated from the exploratory cohort [The Cancer Genome Atlas (TCGA), n = 371)]. HCC cases were classified as high- vs. low-risk groups per overall survival (OS) [hazard ratio (HR) = 2.699]. Significantly, the CCS exhibited great predictive value for prognosis in three independent cohorts, particularly in GSE76427 cohort [area under the curve (AUC) = 0.835/0.822/0.808/0.821/0.826 at 1/2/3/4/5 years]. The nomogram constructed by integrating clinicopathological features with the CCS indicated high accuracy and practicability. Significant enrichment of tumorigenesis-associated pathways was observed in the high-risk patients by Gene Set Enrichment Analysis (GSEA). RT-qPCR revealed that *TICRR* was overexpressed in HCC samples. Increased *TICRR* expression implied poor prognosis in HCC patients. Furthermore, depletion of *TICRR* in HCC cells decreased cell proliferation and the G1/S transition. In conclusion, the established 13-CCS had efficacy in prognostic prediction of HCC patients. Additionally, *TICRR* was demonstrated as a tumor-driver gene for this deadly disease.

## Introduction

Hepatocellular carcinoma (HCC) is ranked as the sixth most common neoplasm throughout the world (https://gco.iarc.fr/). Despite progress being made in the management of HCC, clinical outcomes remain poor (1) partly because clinically ideal biomarkers for specific antitumor decisions are insufficient. Conventional prognostic models, such as the American Joint Committee on Cancer (AJCC) stage (2), still have limited predictive performance. Therefore, there is a need to recognize reliable prognostic markers and anticancer targets for HCC.

The essence of cancer is unlimited cell proliferation mainly caused by misregulation of the cell cycle (3). Cell cycle progression is precisely controlled by checkpoint mechanism and activation states of cyclin-dependent kinases (CDKs) (4, 5). Defects of cell cycle checkpoint and/or hyperactivation of CDKs caused by inactivation of suppressor genes and amplification of oncogenes will result in uncontrolled mitosis. Based on transcriptomic dysregulation, HCC can be divided into two major subgroups: a “proliferation class” and a “non-proliferation class” (6). In contrast to more differentiation of the non-proliferation class, the proliferative HCCs show activation of biological pathways involved in cell cycle regulation and include more aggressive tumors, frequent vascular invasion, and higher levels of serum AFP with worse prognosis (7). Frequently, mutation and upregulation of cell cycle genes are the remarkable molecular features of this class. Subsequent studies have shown that accelerated cell cycle progression contributes to the selection of monoclonal hepatocyte populations and subsequently undergoes genomic alterations that bring about HCC progression (8). Due to the critical role of cell cycle regulation in HCC progression, cell cycle genes may turn into potential biomarkers with optimal clinical practicability in precision medicine of this malignancy.

Through comprehensive analyses of RNA-Seq profiles and clinicopathological data from TCGA cohort, we developed a cell cycle-related 13-mRNA signature (CCS) to provide prognostic information of HCC patients. The predictive precision of the CCS was also measured by two other databases. A nomogram was constructed to assess clinical utility. Concurring with RNA-Seq analysis in TCGA, further functional experiments *in vitro* confirmed that the candidate gene *TICRR* was upregulated in tumor samples and regulated HCC progression.

## Materials and Methods

### Database Analysis

Clinicopathological data and mRNA-seq profiles of HCC patients were obtained from three separate databases. A total number of 371 HCC samples with 50 cases of paired non-cancerous liver tissues from TCGA (https://portal.gdc.cancer.gov; ID: LIHC) were included in the exploratory cohort. Clinicopathological parameters including age, gender, grade, AJCC stage, serum alpha fetoprotein (AFP) value, Child–Pugh grade, vascular invasion (VI) type, and mutation information of TP53 are shown in **Supplementary Material S1**.

Two external validation cohorts GSE76427(n = 115) and LIRI-JP (n = 231) with transcriptomic profiling were extracted from gene expression omnibus (GEO) repository (https://www.ncbi.nlm.nih.gov/geo; Code: GSE76427) and International Cancer Genome Consortium (ICGC) database (https://dcc.icgc.org/projects; Code: LIRI-JP), respectively. Detailed information is exhibited in **Supplementary Material S2**. As necessary, Log2-transformed data of probe-level expression values were used for further analysis.

### Cell Cycle-Related mRNAs and Gene Set Enrichment Analysis

**Figure 1:**
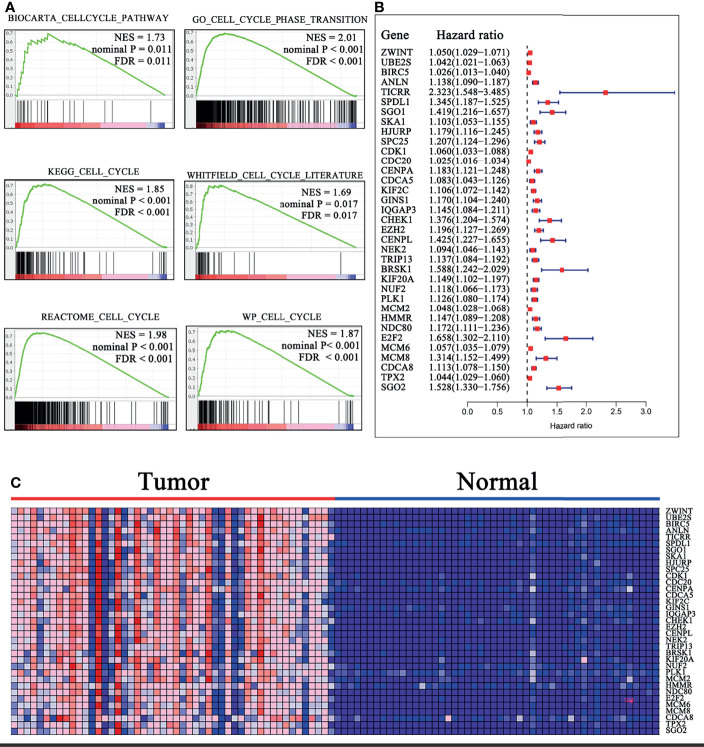
Identification of the cell cycle-related genes. **(A)** Six gene sets were identified to be significantly enriched in HCC samples with FDR <0.25, NES >1.65, and nominal P < 0.02. **(B)** Forrest plot of 35 genes related to OS by univariate Cox analysis in TCGA cohort. P < 0.001. **(C)** Heatmap of 35 OS-related gene expressions in 50 HCC samples relative to normal peritumoral tissues from TCGA cohort. Overexpressed genes are in shades of red, and downregulated genes are in shades of blue. FDR, False discovery rate; NES, Normalized enrichment score; OS, overall survival.

Six cell cycle-related gene sets were obtained from the Molecular Signatures Database (MSigDB; **Figure 1A**). Then, Gene Set Enrichment Analysis (GSEA) was performed to screen out genes that varied at the transcriptome level between cancerous tissue and normal liver epithelium with 1,000 permutations. For each analysis, statistical significance was deemed by false discovery rate (FDR) <0.25, normalized P < 0.05, and |Normalized enrichment score (NES)| >1.65 with 1,000 gene set permutations.

### Construction of the Cell Cycle-Related Gene Signature

Using TCGA cohort, univariate Cox analysis was conducted on the cell cycle-related genes to identify mRNAs associated with OS. Subsequently, these mRNAs were screened out for risk score model construction using a multivariate Cox analysis. The risk score of each sample was calculated by the following formula (Coef: coefficient β; x: value of gene expression):


$$\text{Risk score}=\sum_{\text{i}=1}^{\text{n}}\left(\text{Coef}(\text{i})+\text{x}(\text{i})\right)$$

Median risk score served as the cutoff point to stratify HCC samples into high- or low-risk groups.

### Biological and Functional Analysis

Enrichment of potential pathways in the high-risk group was qualified by GSEA (9). BioCarta (c2.cp.biocarta.v7.2.symbols.gmt), Reactome (c2.cp.reactome.v7.2.symbols.gmt), PID (c2.cp.pid.v7.2.symbols.gmt), and Hallmark (h.all.v7.2.symbols.gmt) gene sets were acquired from the MSigDB database. |NES| >1.65 and normalized P < 0.05 were considered as significant enrichment.

### Establishment of the Nomogram

For quantitative evaluation of prognosis, a nomogram combining the CCS with clinicopathologic characteristics of HCC samples was plotted using the “rms” R package. Calibration analysis and the AUC were used to examine the efficiency of the nomogram.

### Collection of Clinical Samples

Cancerous samples (n = 23) with paired benign lesions (n = 23) were obtained from patients who underwent hepatectomy at the First Affiliated Hospital of Chongqing Medical University from July 2020 to February 2021. Samples were diagnosed as HCC in the Pathology Department of Chongqing Medical University. This research was approved by the Ethics Committee of Chongqing Medical University (Ethical Approval Number: 2021-556). Relevant clinical parameters of the patients, including age, gender, Edmondson–Steiner grade and AJCC stage, serum AFP value, and microvascular invasion (MVI) information, are provided in **Supplementary Material S3**.

### Cell Lines and Transfection

Human HCC cell lines (Hep3B, HepG2, and Huh-7) procured from the Institute of Chinese Academy of Sciences were routinely maintained in our laboratory. All cell lines were authenticated using STR analysis, and the STR profiling report is shown in **Supplementary Material S4.** HCC cells were cultured in Dulbecco’s modified Eagle’s medium (HyClone, Logan, UT, USA) with 10% fetal bovine serum (FBS; Gibco, Australia) and 1% penicillin/streptomycin. Small interfering RNA (siRNA) oligonucleotides targeting *TICRR* were designed and synthetized by GenePharma Biological Technology (China). TICRR-siRNA sequences were as follows: si-TICRR-1, 5′-GGGCCUUCAAGUUCUUUGATT-3′ (sense) and 5′-UCAAAGAACUUGAAGGCCCTT-3′ (anti-sense); si-TICRR-2, 5′-GCCAGCUUCAGGUAUUUCUTT-3′ (sense) and 5′-AGAAAUACCUGAAGCUGGCTT-3′ (anti-sense); si-TICRR-3, 5′-GACCAAAGUUCGAAGAAAUTT-3′ (sense) and 5′-AUUUCUUCGAACUUUGGUCTT′(anti-sense). Cells were transfected with 50 pmol siRNAs by the Lipofectamine 2000 (Invitrogen) for 6 h. Functional tests were performed at 3 days after transfection.

### RNA Isolation and PCR Analysis

Total RNA was extracted from HCC tissues or cell lines using TRIzol reagent (TAKARA, Japan). Reverse transcription of total RNA samples was performed using RT Master Mix for qPCR kit (MCE; No.: HY-K0511). RT-qPCR was performed using ABI Applied Biosystems Prism 7500 Sequence Detection System. Each reaction was conducted with 10-μl mixture containing SYBR Green qPCR Master Mix (MCE; No.: HY-K0501), and glyceraldehyde-3-phosphate dehydrogenase (GAPDH) was used for normalization. Primer sequences are provided in **Supplementary Material S5**.

### Cell Proliferation Assay

Cell viability was detected by the Cell Counting Kit-8 (CCK-8, Biosharp, China) and the 5-ethynyl-2′-deoxyuridine (EdU) kit (RiboBio, C10310-1, China), according to the manufacturer’s instructions. Here, 2,000 cells and 8,000 cells seeded in 96-well plates were used for CCK-8 and EdU prefiltration assay respectively. Images of EdU assay were acquired by a fluorescence microscope (ZEISS, Germany).

### Cell Cycle Analysis

Briefly, HCC cells were fixed in 70% ice-cold ethanol overnight at 4°C and stained with propidium iodide (50 μg/ml). Cell cycle distribution was then detected using a FACSCalibur flow cytometer (BD Biosciences).

### Statistics

All analyses were performed using GraphPad Prism 9 software and R studio 4.0.3. Survival difference was assessed using log-rank test. The variables were presented as the mean ± standard deviation by three separate experiments. For all quantitative analyses, the analyses were blinded, and three observers performed them. P < 0.05 was taken as statistically significant.

## Results

### Development of the Cell Cycle-Related Multi-mRNA Signature

To investigate the cell cycle-related genes that are aberrantly expressed in HCC, we performed a GSEA from the exploratory cohort (TCGA). Six gene sets were identified to be significantly enriched in HCC samples based on FDR <0.25, NES > 1.65, and nominal P < 0.02 (**Figure 1A**). Meanwhile, 907 differentially expressed genes (DEGs) were extracted from these gene sets (CORE ENRICHMENT: YES) for further analysis. Thirty-five genes were discovered to be closely related to OS by univariate Cox analysis (P < 0.001; **Figures 1B, C** and **Supplementary Material S6**). Notably, the 35 mRNAs were all upregulated in the HCC samples relative to normal peritumoral tissues, and *TICRR* (also called Treslin) had the highest hazard ratio (HR; HR = 2.323). Stepwise multivariate Cox analysis was then performed to construct the prognostic signature. Subsequently, 13 cell cycle-related genes (*TICRR*, SPDL1, SGO1, HJURP, CENPA, GINS1, EZH2, BRSK1, NUF2, PLK1, HMMR, E2F2, and CDCA8) were finally developed as an independent indicator of poor prognosis (**Supplementary Material S7**).

### Confirmation of the Prognostic Signature

**Figure 2:**
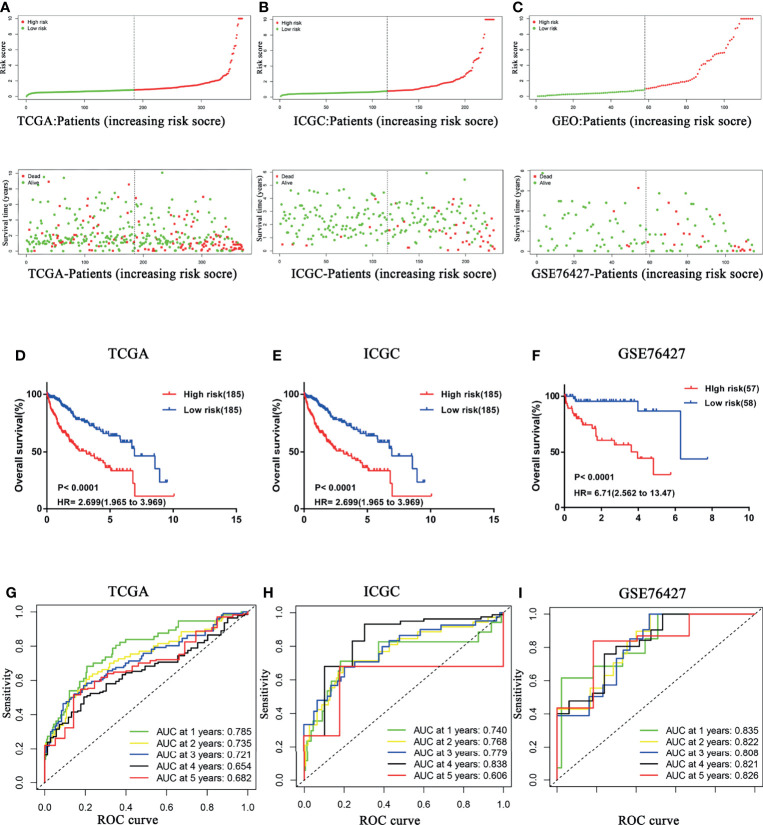
Confirmation of the prognostic signature. HCC samples were dichotomized into two categories according to median risk score in **(A)** TCGA, **(B)** ICGC, and **(C)** GSE76427 cohorts. HCC patients with a low risk score possessed significantly longer OS in **(D)** TCGA, **(E)** ICGC, and **(F)** GSE76427 cohorts. Time-dependent ROC curves of the CCS for prediction of the 1-, 2-, 3-, 4-, and 5-year OS in **(G)** TCGA, **(H)** ICGC, and **(I)** GSE76427 cohorts. OS, overall survival; ROC, Receiver operating characteristic.

With the median risk score of the exploratory cohort as the threshold, all samples were dichotomized into two categories (**Figure 2A**). Kaplan–Meier survival analysis indicated that patients with low risk score possessed significantly longer OS (HR = 2.699, 95% CI 1.965–3.969, P < 0.0001; **Figure 2D**). The predictive efficacy of the CCS was assessed by ROC, and the area under the curve (AUC) values at 1, 2, 3, 4, and 5 years were 0.785, 0.735, 0.721, 0.654, and 0.682, respectively (**Figure 2G**).

To further confirm prognostic accuracy, two other cohorts from ICGC and GEO databases were employed for validation. Both validation cohorts were divided into two groups by the same algorithm aforementioned. Significantly, the high risk score also implied worse survival in both ICGC (**Figures 2B, E**) and GSE76427 (**Figures 2C, F**) cohorts (high risk vs. low risk: HR = 6.85, 95% CI 2.680–9.008; HR = 6.71, 95% CI 2.562–13.47, respectively), consistent with the exploratory cohort described before. Indeed, the model presented a better performance in the two external validation cohorts, especially in GSE76427 cohort (AUC = 0.835/0.822/0.808/0.821/0.826 at 1/2/3/4/5 years, **Figure 2I**). In the ICGC cohort, all the AUC values were more than 0.7 except for the “5-year” (AUC = 0.740/0.768/0.779/0.838/0.606 at 1/2/3/4/5 years; **Figure 2H**). These results above suggested that the predictive power of the CCS had an appropriate sensitivity and specificity.

### The CCS-Based Risk Score Was an Independent Indicator

**Figure 3:**
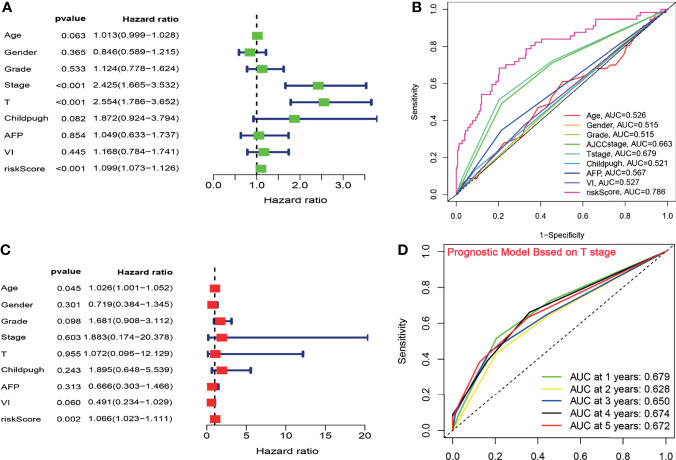
The CCS-based risk score was an independent indicator. **(A, C)** The univariate and multivariate Cox analyses after adjusting for clinical factors. **(B)** ROC curve analysis for the prognostic accuracy of clinicopathological parameters. **(C)** The ROC analysis for several clinicopathological factors and the risk score. **(D)** Time-dependent ROC curves of T stage for prediction of the OS in TCGA cohort. ROC, Receiver operating characteristic; OS, overall survival.

On the basis of the CCS, the univariate and multivariate Cox analyses were performed after adjusting for clinical factors to assess the independence of the risk score in predicting survival. Due to lack of the sample size in M stage (n = 4) and N stage (n = 4), the two parameters were excluded from our analysis. In univariate analysis, the AJCC stage, T stage, and the risk score were observed to be associated with OS (**Figure 3A**). The multivariate analysis suggested that the risk score based on the CCS was a prognostic indicator independent of other parameters (P < 0.001; **Figure 3C**).

To verify the prognostic performance of the risk core with OS, the ROC analysis was further implemented to compare the predictive power of clinicopathological factors with that of the risk score. In the exploratory cohort, the AUC value of the CCS-based risk score reached 0.786, which was higher than that of other clinical factors (**Figure 3B**). Given that T stage had the highest HR from the univariate analysis and the second highest AUC value (AUC = 0.679) from the ROC analysis, we appraised the prognostic accuracy of this factor. The AUC of the signature was larger than that of T stage (signature-AUC = 0.785/0.735/0.721/0.654/0.682 at 1/2/3/4/5 years vs. T stage-AUC = 0.679/0.628/0.650/0.674/0.672 at 1/2/3/4/5 years; **Figure 3D**) in TCGA cohort, demonstrating that the prognostic model could provide better clinical guidance than T stage.

### Stratification Analysis

**Figure 4:**
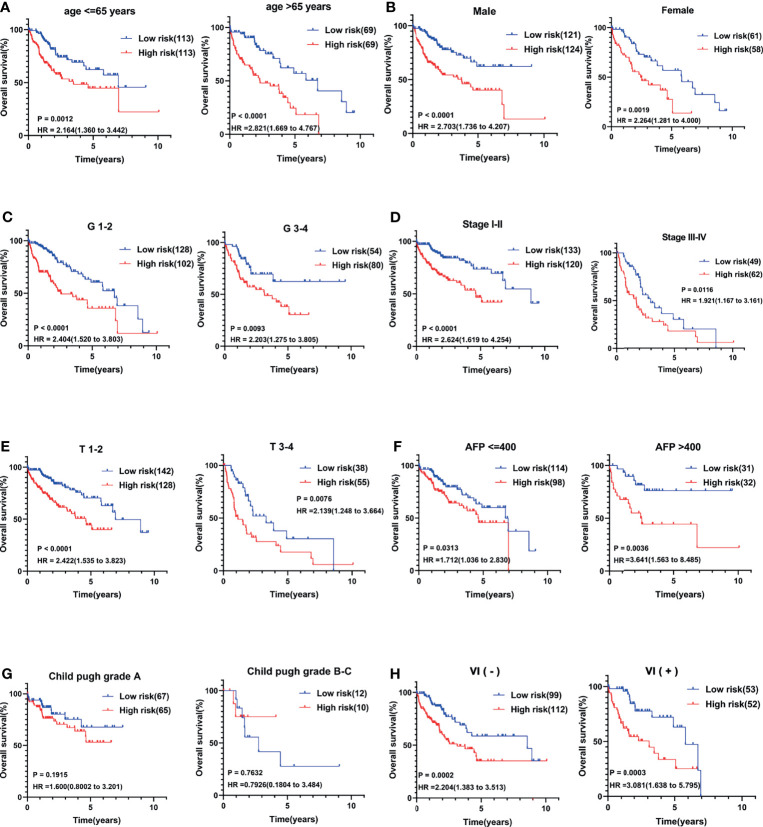
Stratification analysis of several clinical parameters to measure the predictive power of the CCS in each subgroup. Kaplan–Meier curves for association between OS and each subgroup divided by **(A)** age, **(B)** gender, **(C)** grade, **(D)** AJCC stage, **(E)** T stage, **(F)** AFP value, **(G)** Child–Pugh grade, and **(H)** vascular invasion (VI) status. OS, overall survival; AJCC stage, American Joint Committee on Cancer stage.

Several clinical parameters were stratified to measure the predictive power of the CCS in each subgroup. The 13-CCS had no OS-predictive power for the two subgroups of Child–Pugh grade (P > 0.05; **Figure 4G**). However, high risk score presented shorter survival in the other subgroups, including age ≤65 years (HR = 2.164), age >65 years (HR = 2.821; **Figure 4A**), men (HR = 2.703), women (HR = 2.264; **Figure 4B**), G1–2 (HR = 2.404), G3–4 (HR = 2.203; **Figure 4C**), stages I–II (HR = 2.624), stages III–IV (HR = 1.921; **Figure 4D**), T1–2 (HR = 2.422), T3–4 (HR = 2.139; **Figure 4E**), AFP ≤400 (HR = 1.712), AFP >400 (HR = 3.641; **Figure 4F**), VI(-) (P = 0.0002), and VI (+) (HR = 3.081; **Figure 4H**). Stratification analysis of the 13-mRNA model further verified that the CCS predicted the OS with precision in division of these parameters.

### The CSS Was Correlated With HCC Progression

**Figure 5:**
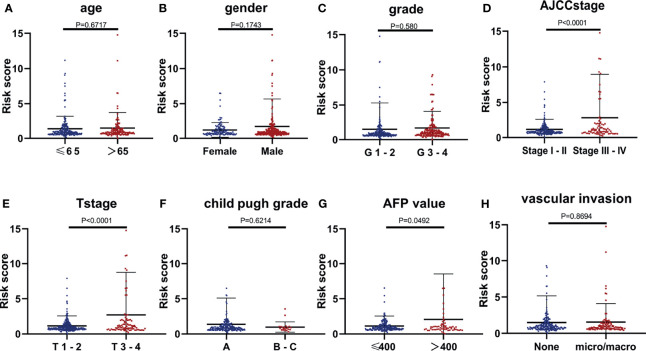
The relevance of the CCS-based risk score to each clinical feature. **(A)** Age (P = 0.6717), **(B)** gender (P = 0.1743), **(C)** grade (P = 0.580), **(D)** AJCC stage (P < 0.0001), **(E)** T stage (P < 0.0001), **(F)** Child–Pugh grade (P = 0.6214), **(G)** AFP value (P = 0.0492), and **(H)** vascular invasion (VI; P = 0.8694).

The relevance of the CCS-based risk score to each clinical feature was evaluated. Stratification was performed according to the same method, and the results suggested that the risk score had no association with age, gender, grade, Child–Pugh grade, and VI of patients. However, HCC samples in advanced clinicopathological stage (Stages III–IV or T3–4, P < 0.001) and high level of AFP (AFP >400, P = 0.0492) were positively correlated with the risk score (**Figures 5A–H**), reflecting that the CCS-based risk score was involved in HCC progression.

**Figure 6:**
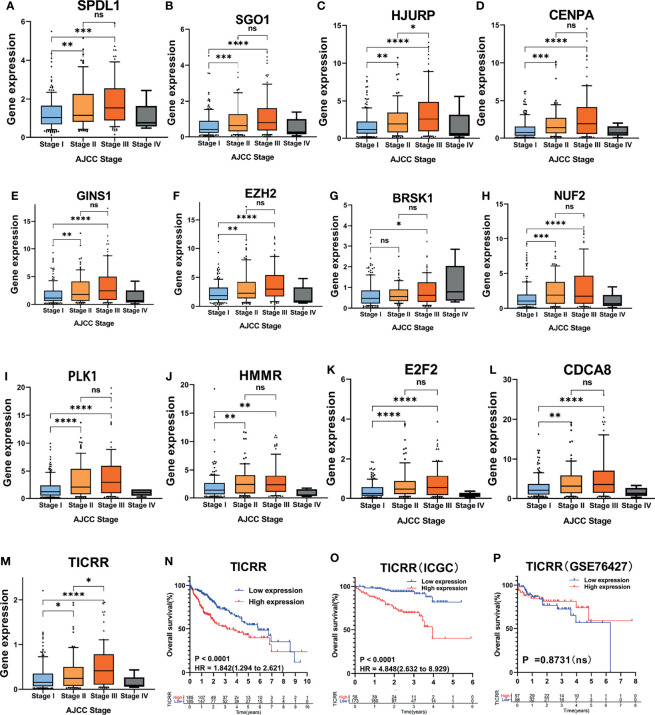
Association of the 13 cell cycle-based genes with AJCC stage in TCGA cohort. **(A–M)** Expression of 13 cell cycle-based mRNAs in HCC patients with different AJCC stages: Stage I (n = 170), Stage II (n = 85), Stage III (n = 85), and Stage IV (n = 4) from TCGA cohort. *P < 0.05, **P < 0.01, and ***P < 0.001. NS, nonsignificant. **(N–P)** Kaplan–Meier curves for OS of HCC patients plotted against time (years) based on *TICRR* expression levels from TCGA cohort (n = 371), ICGC cohort (n = 231), and GSE76427 (n = 115). ****P < 0.0001, OS, overall survival.

Besides, analysis of distinct expression of the 13 genes among different AJCC stages in HCC samples showed that the expression of the 13 mRNAs was positively correlated with AJCC stage (**Figures 6A–M**). Similarly, because the sample size in Stage VI (n = 4) was too small, we did not count it for statistics. Almost all the 13 genes were upregulated in later AJCC stage, such as stage III vs. Stage I (P < 0.05) and Stage II vs. Stage I (except BRSK1, P < 0.05). Interestingly, only *TICRR* and HJURP expression had a difference between Stage II and Stage III (**Figures 6C, M**). In addition, through Kaplan–Meier curve analysis, a higher *TICRR* level resulted in significantly worse prognosis in TCGA datasets (HR = 1.842; **Figure 6N**) and ICGC datasets (HR = 4.848; **Figure 6O**), while for the GSE76427, no significant association with survival was found (**Figure 6P**).

### Establishment of the Nomogram

**Figure 7:**
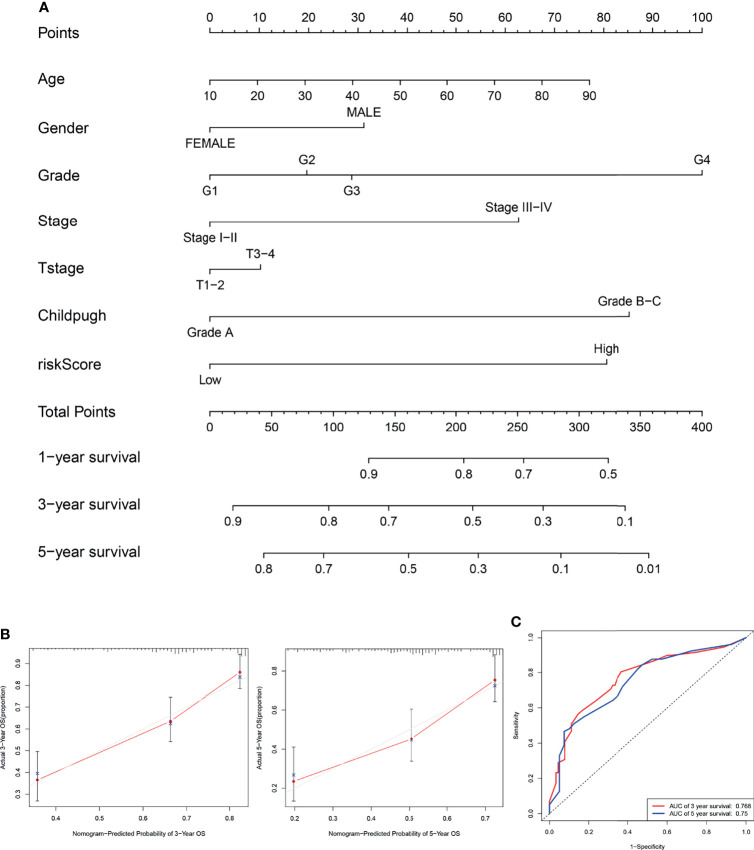
Establishment of the nomogram. **(A)** The nomogram integrating clinicopathological parameters and the CCS-based risk score for predicting the 3- and 5-year OS of HCC patients. **(B)** Calibration plots for both 3-year OS and 5-year OS in ideal prediction with actual observations. **(C)** The ROC curves for evaluating the prognostic performance of the nomogram for 3-year OS and 5-year OS. OS, overall survival.

The nomogram integrating clinicopathological parameters and the CCS-based risk score was developed to provide a clinical usability of the prognostic model (**Figure 7A**). Calibration plots for both 3-year OS and 5-year OS confirmed considerable coherence of ideal prediction with actual observations (**Figure 7B**). AUC at 3-year OS and 5-year OS reached 0.768 and 0.75, respectively (**Figure 7C**), showing the robustness and accuracy of the nomogram.

### Biological and Functional Analysis

**Figure 8:**
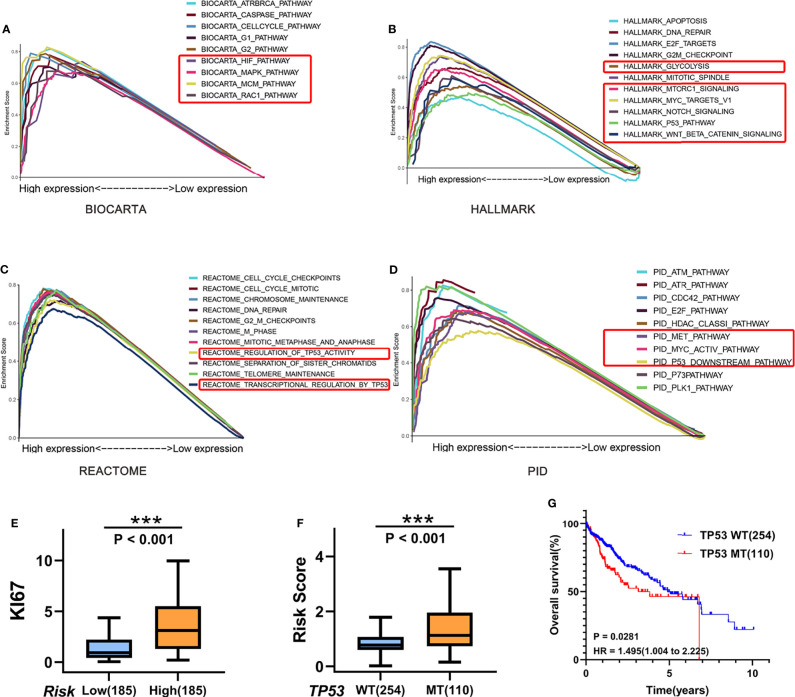
Biological and functional analysis. **(A–D)** Pathway analysis in the high-risk group using **(A)** BIOCARTA, **(B)** Hallmark, **(C)** REACTOME, and **(D)** PID gene sets by GSEA. **(E)** Boxplots of KI67 gene expression in the high-risk (n = 254)/low-risk (n = 110) group in TCGA cohort. **(F)** Boxplots show the differences of the risk score between TP53-wild type (n = 254) and TP53-mutant (n = 110) for HCC patients in TCGA cohort. **(G)** Kaplan–Meier curves for correlation of OS with TP53 mutation status for HCC in TCGA cohort. ***P < 0.001.

To obtain mechanistic grasp of the cell cycle-related processes in biological functions, we performed a pathway analysis in the high-risk group using BIOCARTA, Hallmark, REACTOME, and PID gene sets by GSEA. As shown in **Figures 8A–D**, several canonical pathways, such as MCM, MAPK, Glycolysis, Wnt, and Myc pathways, which associated with proliferation/aggressiveness phenotype, were enriched in the high-risk samples. Consistent with this, the high-risk group had higher expression of the proliferation marker gene “KI67” than that in the low-risk group in TCGA cohort (P < 0.001; **Figure 8E**). Given that patients of the “proliferation class” usually have more frequent TP53 mutations and worse outcomes, we further examined the relationship among these factors. As expected, patients with TP53 mutations have a higher risk score (P < 0.001) and worse prognosis (HR = 1.495) (**Figures 8F, G**).

### Cell Cycle Progression Analyses of the CCS

**Figure 9:**
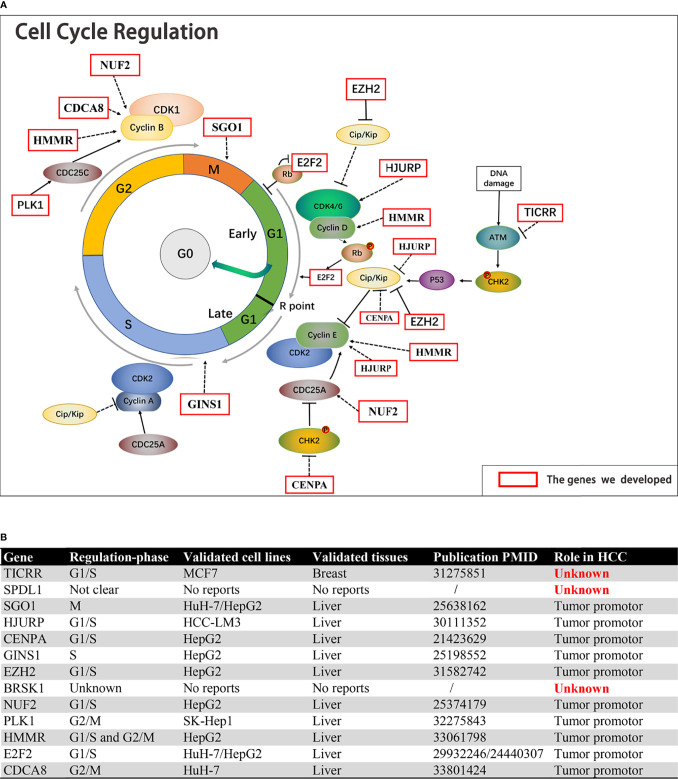
The function of the 13 genes in cell cycle regulation and their roles in HCC. **(A, B)** Ten of the 13 cell cycle genes, including SGO1, HJURP, CENPA, GINS1, EZH2, NUF2, PLK1, HMMR, E2F2, and CDCA8, were established HCC driver genes. TICRR, SPDL1, and BRSK1 had no confirmed role in HCC.

The function of the 13 genes in cell cycle regulation and their roles in HCC were analyzed. Ten of the 13 cell cycle genes, including SGO1 (10), HJURP (11), CENPA (12), GINS1 (13), EZH2 (14), NUF2 (15), PLK1 (16), HMMR (17), E2F2 (18, 19), and CDCA8 (20), have been confirmed by previous studies, indicating that their overexpression could increase the malignant phenotype of HCC (**Figures 9A, B**). Five of these have been identified as activators for G1/S transition in HCC cells, including HJURP, CENPA, EZH2, NUF2, and E2F2. GINS1 and SGO1 regulated S-phase and M-phase duration respectively. HMMR participated in both G1/S and G2/M transitions (17), while elevated E2F2 or PLK1 activated G2/M transitions in HCC cells. However, *TICRR*, SPDL1, and BRSK1 had no confirmed role in HCC.

### *TICRR* Was Related to HCC Prognosis and Tumor Progression

**Figure 10:**
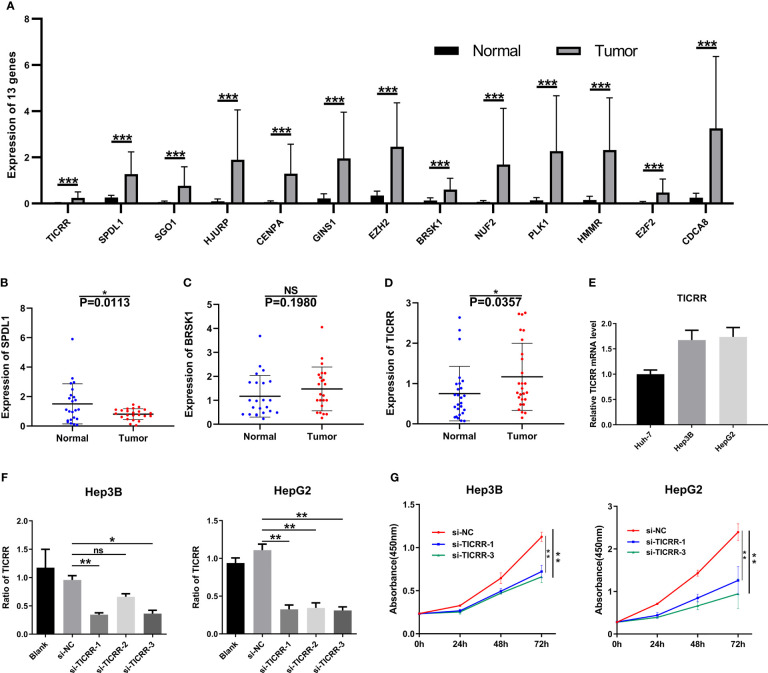
Expression verification and functional experiments. **(A)** mRNA levels of the 13 genes in the HCC samples (n = 50) compared to normal peritumoral tissues (n = 50) in TCGA cohort. Expression of **(B)** SPDL1, **(C)** BRSK1, and **(D)** *TICRR* in HCC tissues (n = 23) compared to normal peritumoral tissues (n = 23), as measured by RT-qPCR. **(E)** Expression of *TICRR* in HUH-7, Hep3B, and HepG2 cells, as measured by RT-qPCR. **(F)** Knockdown efficiency of *TICRR* in Hep3B and HepG2 using three independent siRNAs. **(G)** CCK-8 analysis showed that depletion of *TICRR* in Hep3B and HepG2 cells by 2 separate siRNAs led to decreased cell proliferation. *P < 0.05, **P < 0.01, and ***P < 0.001. NS, nonsignificant.

All 13 genes were all strikingly overexpressed in the HCCs relative to adjacent non-neoplastic tissues in TCGA cohort (P < 0.001; **Figure 10A**), and detailed gene expression data are available in **Supplementary Material S8**. RT-qPCR was performed to further detect the expression of the three function-unknown genes. SPDL1 expression was decreased in HCCs compared with non-neoplastic liver tissues (**Figure 10B**). The expression of BRSK1 mRNA tended to be higher in HCC relative to paired normal liver tissue, but without reaching statistical significance (P = 0.198; **Figure 10C**). Importantly, we observed that expression of *TICRR* mRNA was elevated in HCCs relative to respective controls (P < 0.05; **Figure 10D**), consistent with TCGA RNA-seq findings. We also noticed that *TICRR* at mRNA level was negatively correlated with OS in HCC patients (**Figures 6N, O**), suggesting its potentially important role in tumor progression.

**Figure 11:**
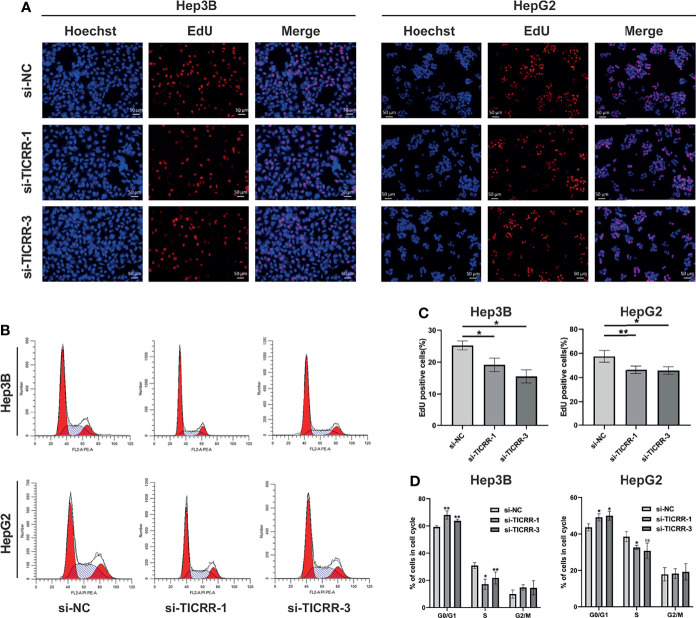
Downregulation of *TICRR* attenuated proliferation by promoting G1/S arrest. **(A, C)** Knockdown of *TICRR* in Hep3B and HepG2 cells by both 2 separate *TICRR* siRNAs caused decreased proliferation, as measured by EdU assay. **(B, D)** Silencing *TICRR* restrained G1/S transition in Hep3B and HepG2 cells by two independent si-*TICRRs* in the cell cycle analysis. *P < 0.05, **P < 0.01, and ***P < 0.001. NS, nonsignificant.

After comparing *TICRR* expression in three HCC cells (**Figure 10E**), HepG2 and Hep3B cells were chosen for transfection with three independent siRNAs targeting *TICRR*. Based on knockdown efficiency, two siRNAs (si-TICRR-1 and si-TICRR-3) were employed in depletion of *TICRR* (**Figure 10F**). The effect of *TICRR* on HCC cell proliferation was studied by CCK-8 (**Figure 10G**) and EdU assays (**Figures 11A, C**), and the results demonstrated that depletion of TICRR in Hep3B and HepG2 cells by 2 separate *TICRR* siRNAs led to decreased cell proliferation. In addition, the cell cycle analysis revealed that silencing *TICRR* restrained G1/S transition in Hep3B and HepG2 cells by 2 independent si-TICRRs (**Figures 11B, D**). Taken together, downregulation of *TICRR* attenuated proliferation by promoting G1/S arrest in HCC cells.

## Discussion

Cancer arises from several molecular alterations that drive uncontrolled proliferation. Loose of the cell cycle regulation is central to this oncogenic proliferation, and dysregulation in markers (such as E2F1, PLK1, CCNE1, and CCND1) associated with cell cycle mechanism is one of the most prominent molecular aberrations in all cancers (21). Genomic studies based on RNA sequencing has identified 2–6 major alterations in several cellular pathways. They are considered as functional “driver” alterations that alter key signaling pathways leading to HCC progression, and cell cycle regulation is one of them. Recent studies have uncovered abundant genetic alterations in the cell cycle pathway. Several cell cycle-regulated genes are involved in liver carcinogenesis. For instance, the mutation rates of TP53, ATM, CDKN2A, CCNE1, and RB1 reached 2%–48%, 2%–6%, 2%–9%, 5%, and 3%–8%. Besides, mutations of these genes are closely correlated with a poorer clinical outcome in patients treated with liver resection (22). Different prognoses between the nonproliferation class and proliferation class may be caused by different activation states of pro-survival pathways. The proliferative HCCs include clinically more aggressive tumors with frequent VI and is characterized by enrichment in frequent activations of cell cycle pathways including TP53 inactivating mutations and amplification of CCND1. However, due to the intratumor heterogeneity of HCC (23), the malignant transformation of liver cannot be fully explained by alternations in one or several particular signaling molecules (e.g., KRAS, BRAF, MEK, and ERK) and/or oncogenic pathways (e.g., AKT/mTOR, MAPK, and Wnt/β-catenin). Notably, all these pathways converge on the point of the cell cycle regulation, facilitating cells through the restriction point and activating the G1/S transition (3). Therefore, as a prominent hallmark of cancer malignancy and an integration point for upstream signaling pathways, cell cycle-related genes are potential biomarkers of prognosis and therapeutic targets with clinical value. In practice, several cell cycle-directed biomarkers for prognosis evaluation have been developed with powerful prognostic performance. For example, the more aggressive subgroup of HCC “G3” consists of TP53 mutations and upregulation of genes that regulate the cell cycle, acting as an independent predictor of cancer recurrence (24). As part of pre-replicative complexes (pre-RCs) that enable G1/S transition, Mcm2–7 are effective prognostic indicators in various kinds of malignancies (e.g., non-small-cell lung cancer) (25).

**Figure 12:**
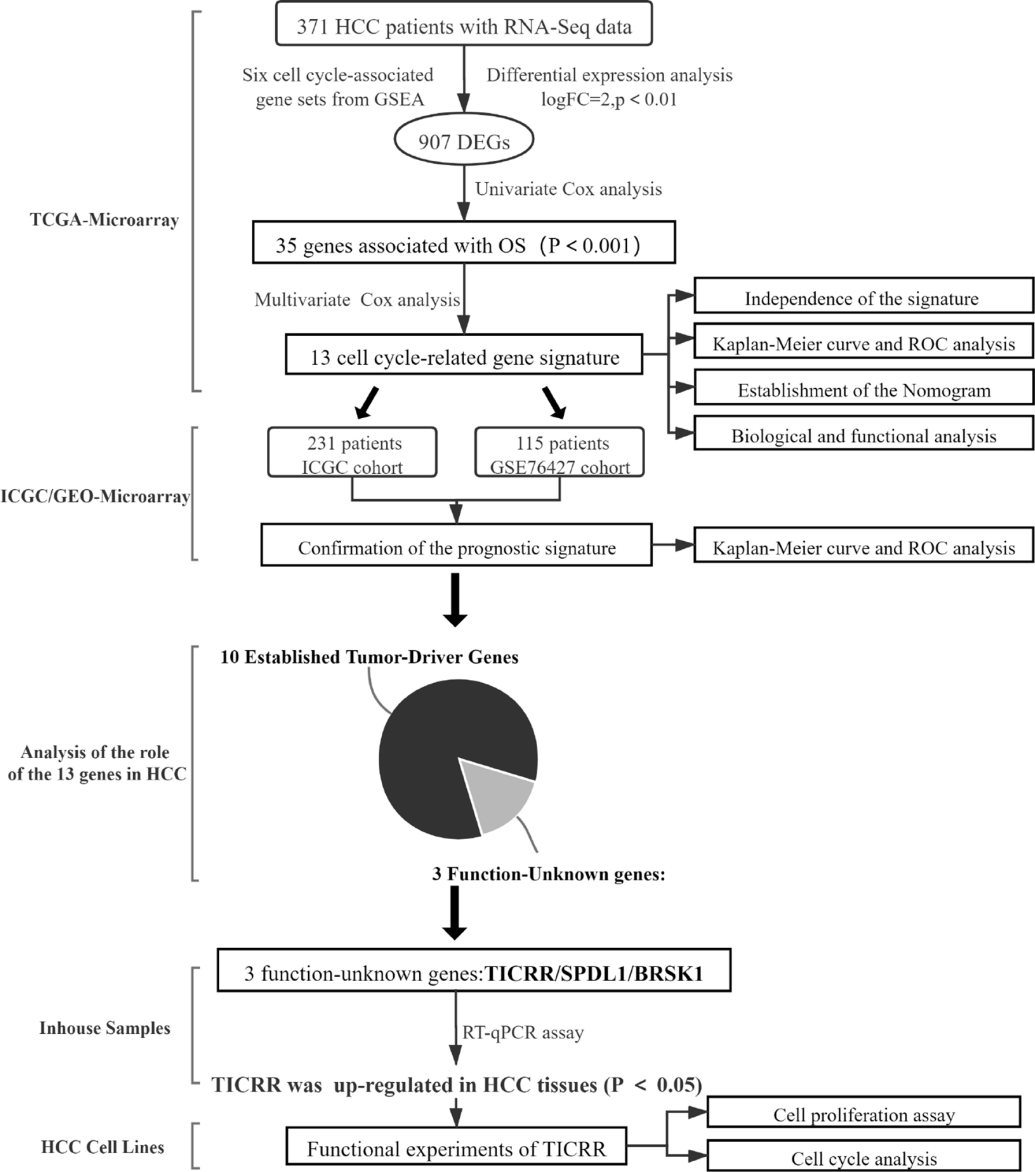
A workflow diagram of this research.

Through comprehensive analyses of the clinicopathological data and transcriptomic profiles from public sources, we established a cell cycle-related 13-mRNA signature for progression assessment of HCC patients. A workflow diagram of this study, as provided in **Figure 12**, displayed the overall process of data analysis and method implementation. Importantly, based on TCGA cohort, we used two independent datasets as external validation. Significantly, the AUC values of the signature in GSE76427 cohorts at 1, 2, 3, 4, and 5 years were all more than 0.8, showing high accuracy in survival prediction of the HCC sample. Further GSEA showed that tumor progression/recurrence-associated pathways (e.g., MCM, MAPK, Glycolysis, Wnt, Myc, and p53) were evidently enriched in the high-risk patients with HCC. Consistent with our results, cell cycle regulation pathways (e.g., E2F, PLK1, and ATM) were also enriched in these patients. Deregulated Myc and E2F expression has also been also found to prevent normal differentiation. Several cell cycle-related multi-gene models have shown appropriate capacity in prognosis prediction for diverse tumor types. Based on eight cell cycle-immunity-related genes, Chen et al. (26) constructed a prognosis model to predict the OS in Lung adenocarcinoma (LUAD) samples, and this model was correlated with immune cell infiltration. Similarly, using bioinformatics analysis, another concurrent study has shown that a G2/M checkpoint-related signature composed of five genes (MARCKS, CCNF, MAPK14, INCENP, and CHAF1A) was connected with the prognosis of gastric cancer (GC) patients (27). A G2/M pathway score was closely related to aggressive characteristics and prognosis in patients with estrogen receptor (ER)-positive breast cancer (28). Importantly, Shariat et al. (29) have confirmed that a multi-biomarker risk model outstrips single molecule in precisely predicting disease prognosis. This research has shown that cell cycle-related biomarkers used for predictive purposes may have an increased clinical use in the near future. Consistent with those studies, the cell cycle signature composed of 13 cell cycle-related genes (*TICRR*, SPDL1, SGO1, HJURP, CENPA, GINS1, EZH2, BRSK1, NUF2, PLK1, HMMR, E2F2, and CDCA8) possessed superior specificity and sensitivity in predicting the OS of HCC patients. Furthermore, we debated the roles of 13 genes in HCC, 10 of these are established HCC driver genes, including SGO1, HJURP, CENPA, GINS1, EZH2, NUF2, PLK1, HMMR, E2F2, and CDCA8. For example, PLK1 played a pivotal role in various aspects of mitosis, emerging as a promising marker for prognosis determination (30). At present, PLK1-targeted medicine is undergoing clinical trials for multiple types of solid tumors. We also found that three genes (BRSK1, *TICRR*, and SPDL1) in the signature had no clarified role in HCC. For verification purposes, we measured the mRNA expression of these genes in our HCC samples compared with public transcriptomic data of HCC samples and found that *TICRR* was generally upregulated in HCC tissues. Unexpectedly, contrary to TCGA cohort data, decreased SPDL1 mRNA was observed in our HCC samples. SPDL1 (also referred to as Spindly/CCDC99), a recently identified regulator of mitosis, takes part in mitotic spindle formation and chromosome segregation (31). Kodama et al. (32) have shown that SPDL1 was a human CRC tumor-suppressor gene, and decreased SPDL1 was related to shorter survival in CRC. However, higher SPDL1 expression indicated a poorer prognosis (HR = 1.345) in HCC patients from TCGA cohort.

*TICRR* is a pivotal DNA replication modulator that regulates the progression of S phase (33). Yu et al. (34) have shown that *TICRR* was significantly elevated in diverse tumor types in *in silico* analysis. In addition, depletion of *TICRR* suppressed breast cancer cell viability and caused cell cycle arrest at G1 phase by activating DNA damage response and p53 signaling pathway (34). In the context of our results, among the 35 genes associated with OS, *TICRR* had the highest HR (HR = 2.323, P < 0.0001). *TICRR* expression had statistical difference in diverse AJCC stages (stage I vs. stage II, stage II vs. stage III, and stage I vs. stage III) in TCGA cohort, and its high expression meant a poorer prognosis in both TCGA and ICGC cohorts. Subsequent experiments proved that knocking down of *TICRR* inhibited proliferation and promoted G1/S arrest in HCC cells. All these indicated that *TICRR* may be a potential indicator for prognosis prediction and a promising anticancer target.

In fact, our research had certain limitations. Our analysis was a retrospective study that could cause a selection bias. The predictive model, therefore, still needed more explicit evidence for clinical usage. Moreover, the specific mechanism of *TICRR* and SPDL1 in modulating cell cycle division deserved further study. Collectively, the signature composed of 13 cell cycle-related genes had a favorable effect in predicting the survival of HCC patients. Besides, high levels of *TICRR* were associated with advanced clinicopathological stage and poor prognosis in HCC. Downregulation of *TICRR* attenuated proliferation by triggering G1/S arrest in HCC cells by *in vitro* experiments. These results provided valuable insights into the individualized management of this deadly disease.

## Data Availability Statement

The datasets presented in this study can be found in online repositories. The names of the repository/repositories and accession number(s) can be found in the article/**Supplementary Material**.

## Ethics Statement

The studies involving human participants were reviewed and approved by the Ethics Committee of Chongqing Medical University. The patients/participants provided their written informed consent to participate in this study.

## Author Contributions

YZ and FL contributed to study design, data analysis, and article writing. Y Zhou and F Luo performed the experiments. D-LL and G-LH provided samples and clinical information. YZ, D-LL, and G-LH contributed equally to reviewing the article before submission. All authors contributed to the article and approved the submitted version.

## Funding

This study was funded by the National Natural Science Foundation of China (grant number 81372481).

## Conflict of Interest

The authors declare that the research was conducted in the absence of any commercial or financial relationships that could be construed as a potential conflict of interest.

## Publisher’s Note

All claims expressed in this article are solely those of the authors and do not necessarily represent those of their affiliated organizations, or those of the publisher, the editors and the reviewers. Any product that may be evaluated in this article, or claim that may be made by its manufacturer, is not guaranteed or endorsed by the publisher.
