# Supplementary material for: A Cell Cycle-Related 13-mRNA Signature to Predict Prognosis in Hepatocellular Carcinoma
Source: Front Oncol. 2022 Mar 28;12:760190. doi: 10.3389/fonc.2022.760190 (PMC8995863; doi:10.3389/fonc.2022.760190)
Supplement: Supplementary file 1 [file DataSheet_1.zip › Supplementary File S4. HCC cell lines-STR profiling report.pdf]

Supplementary File S4. Cell Line Authentication- STR Profiling Report		
Cell Line	Authentication institution	The region where the institution belongs
HepG2	Zhong Qiao Xin Zhou Biotechnology Co, Ltd	Shanghai, China
Hep3B	Tsingke Biological Technology Co, Ltd	Hunan, China
Huh-7	Zhong Qiao Xin Zhou Biotechnology Co, Ltd	Shanghai, China

HepG2-STR Profiling Report

中乔新舟
CELL RESEARCH

Cell Line Authentication – STR Profiling Report

Sample Type: Cell Line
Testing Type: STR

Sample code:

Table 1. Sample Code

Customer's code	Company Code
632	20200102-02

Sample Number:1

Sample Type: Cell line

Testing Type: STR

Sample From: Shanghai Zhong Qiao Xin Zhou Biotechnology Co.,Ltd.

Testing Method:

DNA was extracted by a commercial kit from CORNING (AP-EMN-BL-GDNA-250G). Twenty short tandem repeat (STR) loci plus the gender determining locus, Amelogenin, were amplified by six multiplex PCR and separated on ABI 3730XL Genetic Analyzer. The signals were then analyzed by the software GeneMapper.

Data Interpretation:

Cell lines were authenticated using Short Tandem Repeat (STR) analysis as described in 2012 in ANSI Standard (ASN-0002) by the ATCC Standards Development Organization (SDO) and in Capes-Davis et al., Match criteria for human cell line authentication: Where do we draw the line? Int J Cancer. 2013;132(11):2510-9.

HepG2-STR Profiling Report

中乔新舟
CELL RESEARCH

Certificate of STR Analysis

Test Results:

1. Result

Table 2. Matching information on the cell lines

Sample Code	Multi-allele	Cell line matched	Cell Bank	EV
20200102-02	No	Hep-G2	DSMZ	0.94

- Multi-allele means some STR contain more than two loci.

2. Sample Description

20200102-02:

A. The STR results showed that no multiple alleles were found in this cell line, and no cross contamination of human cells was found in the cell line.

B. The DNA of the cell lines found to match the type of cell lines basically in a cell line retrieval, DSMZ database shows that cells called **HEP-G2,** corresponding to the cell number **180.**

3. Genotyping Result

Table 3. STR and Amelogenin Genotyping Results of Cell line 20200102-02

Loci	Sample information			Cell Bank information		
	Sample name：632			Cell line name：HEP-G2		
	Allele1	Allele2	Allele3	Allele1	Allele2	Allele3
D5S818	11	12		11	12	
D13S317	9	13		9	13	
D7S820	10	10		10	10	
D16S539	12	12		12	13	
VWA	17	17		17	17	

HepG2-STR Profiling Report

中乔新舟
CELL RESEARCH

Certificate of STR Analysis

TH01	9	9	9	9
AMEL	X	Y	X	Y
TPOX	8	9	8	9
CSF1PO	10	11	10	11
D12S391	21	25		
FGA	22	25		
D2S1338	19	20		
D21S11	29	31		
D18S51	13	14		
D8S1179	15	16		
D3S1358	15	16		
D6S1043	13	13		
PENTAE	15	20		
D19S433	15.2	15.2		
PENTAD	9	13		
D1S1656	11	12		

The allele match algorithm compares the 8 core loci plus amelogenin only, even though alleles from all loci will be reported when available.

HepG2-STR Profiling Report

中乔新舟
CELL RESEARCH

Certificate of STR Analysis

Others:

1. Genotyping Strategy and Site Distribution

Attached Table. Experimental Strategy and Sites

	**Strategy 1**	**Strategy 2**	**Strategy 3**	**Strategy 4**
1	D3S1358	D8S1179	D19S433	AMEL
2	VWA	D21S11	TH01	D1S1656
3	D7S820	D16S539	D13S317	D5S818
4	CSF1PO	D2S1338	TPOX	D12S391
5	PENTAE	PENTAD	D18S51	FGA
6			D6S1043	

2. DSMZ tools was used to carry on the cell line comparison, which contains 2455 cell lines STR data from ATCC, DSMZ, JCRB, ECACC, GNE and RIKEN databases. If the cell is not included in the above cell library, users need to compared with other databases.

HepG2-STR Profiling Report

中乔新舟
CELL RESEARCH

Certificate of STR Analysis

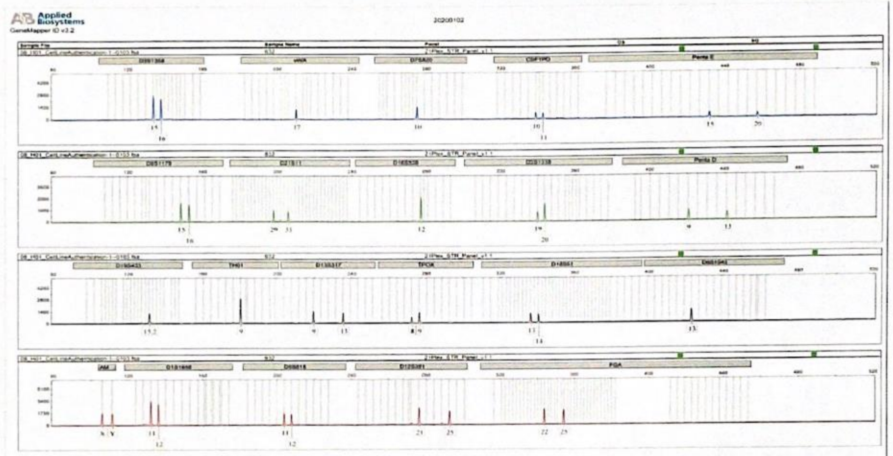

Report Date:
Jan., 2020

Hep3B-STR Profiling Report

TSINGKE Biological Technology

湖南擎科生物　　提供优质、快速 DNA 测序和合成服务以及领域内专业分子试剂

cs-seq@tsingke.net(测序) cs-synth@tsingke.net (合成)

检 测 结 果

检验基本情况

样品编号	多等位基因	匹配细胞系	细胞库	EV 值	匹配说明
**HEP3B2-1-7**	无	HEP-3B	DSMZ	0.94	基本匹配

- 多等位基因指三等位及以上基因现象。

样本描述

- HEP3B2-1-7：该株细胞 DNA 分型在细胞系检索中找到 **基本匹配** 的细胞系，DSMZ 数据库显示细胞名为 HEP-3B。本次检测在该细胞系中**无多等位基因**。

备注：待测细胞系与收录于 ATCC, DSMZ, JCRB 和 RIKEN 数据库的细胞系 STR 数据进行比对，未收录于以上细胞库的细胞系将无法匹配。

Hep3B-STR Profiling Report

TSINGKE
Biological Technology

湖南擎科生物　　提供优质、快速 DNA 测序和合成服务以及领域内专业分子试剂

cs-seq@tsingke.net(测序) cs-synth@tsingke.net (合成)

样本分型结果详情

细胞 HEP3B2-1-7 的 STR 位点和 Amelogenin 位点的基因分型结果						
	送检细胞 STR 信息			细胞库细胞 STR 信息		
	送检细胞名：HEP3B2-1-7			细胞库细胞名：HEP-3B		
Loci	Allele1	Allele2	Allele3	Allele1	Allele2	Allele3
D5S818	13	13		13	13	
D13S317	12	14		12	14	
D7S820	8	10		8	10	
D16S539	10	10		10	10	
VWA	16	17		17	17	
TH01	6	7		6	7	
AMEL	X	X		X	X	
TPOX	9	9		9	9	
CSF1PO	8	8		8	8	
D12S391	17	17				
FGA	18	18				
D2S1338	21	25				
D21S11	30	31				
D18S51	20	20				
D8S1179	12	12				
D3S1358	15	15				
D6S1043	12	17				
PENTAE	5	16				
D19S433	12.2	14				
PENTAD	12	14				

感谢您对湖南擎科的信任！

湖南擎科技术部
17788901165
20180502

Hep3B-STR Profiling Report

TsingKe Biological Technology 177-8890-1165 cs-seq@tsingke.net cs-synth@tsingke.net

Cell Line Authentication Service

Sample information

1. Sample name

HEP3B2-1-7

2 Sample type

Cell suspension

experimental method

1. Extraction of Cell Genomic DNA

Genomic DNA. extraction of cells using Tsingke's Animal Genome extraction Kit (TSP201-200)

2. Amplification using 20 locus specific fluorescent primers

Use Tsingke's gold Mix (green) (number TSE101) to amplify.

experimental result

Sample number	multiple alleles	Matched cell line	Cell library	EV value	Matching description
HEP3B2-1-7	NO	HEP-3B	DSMZ	0.94	BASIC MATCH

- *Polyalleles refer to the third class and above gene phenomenon.*

Note: the cell line to be tested and recorded in the ATCC, DSMZ, JCRB and RIKEN database of cell line STR data, the cell line not included in the above cell bank will not match.

Hep3B-STR Profiling Report

177-8890-1165 cs-seq@tsingke.net cs-synth@tsingke.net

Details of sample typing results

Genotyping Result of STR and Amelogenin loci in HEP3B2-1-7 cells						
	Cell STR information			Cell library cell STR information		
	Cell name:HEP3B2-1-7			Cel libraryl name:HEP-3B		
Loci	Allele1	Allele2	Allele3	Allele1	Allele2	Allele3
D5S818	13	13		13	13	
D13S317	12	14		12	14	
D7S820	8	10		8	10	
D16S539	10	10		10	10	
VWA	16	17		17	17	
TH01	6	7		6	7	
AMEL	X	X		X	X	
TPOX	9	9		9	9	
CSF1PO	8	8		8	8	
D12S391	17	17				
FGA	18	18				
D2S1338	21	25				
D21S11	30	31				
D18S51	20	20				
D8S1179	12	12				
D3S1358	15	15				
D6S1043	12	17				
PENTAE	5	16				
D19S433	12.2	14				
PENTAD	12	14				

Key experimenter: He Liangbo

Reviewer: He Liangbo

Responsible person: He Liangbo

Issue date: 02 MAY 2018

Huh-7-STR Profiling Report

中乔新舟
CELL RESEARCH

Certificate of STR Analysis

细胞遗传质量鉴定检验报告

检品名称：细胞系

检验类型：STR基因型检验

样品编号：

表1 样本编号

客户样本编号	公司编号
372	20171213-07

样品数量：1

样品性状：细胞系

检测项目：STR

送检单位：上海中乔新舟生物技术有限公司

检测方法：用 Axygen 的基因组抽提试剂盒提取 DNA，采用 20- STR 扩增方案扩增，在 ABI 3730XL 型遗传分析仪上对 STR 位点和性别基因 Amelogenin 进行检测。

检验结果：

(一)检验基本情况

表2：样本基因型检验结果

	多等位基因	匹配细胞系	细胞库	EV值	匹配说明
20171213-07	无	HuH-7	DSMZ	1.00	完全匹配

- 多等位基因指三等位及以上基因现象。
- 本次检测各细胞分型结果良好。

Huh-7-STR Profiling Report

中乔新舟
CELL RESEARCH

Certificate of STR Analysis

(二)各样本描述

- 20171213-07：该株细胞 DNA 分型在细胞系检索中找到**完全匹配**的细胞系，DSMZ 数据库显示细胞名为 **HUH-7**，细胞号对应 **JCRB0403**。本次检测在该细胞系中**没有发现多等位基因。**

(三)样本分型结果

细胞 20171213-07 的 STR 位点和 Amelogenin 位点的基因分型结果						
Loci	送检细胞 STR 信息			细胞库细胞 STR 信息		
	送检细胞名：372			细胞库细胞名：HuH-7		
	Allele1	Allele2	Allele3	Allele1	Allele2	Allele3
D5S818	12	12		12	12	
D13S317	10	11		10	11	
D7S820	11	11		11	11	
D16S539	10	10		10	10	
VWA	16	18		16	18	
TH01	7	7		7	7	
AMEL	X	X		X	X	
TPOX	8	11		8	11	
CSF1PO	11	11		11	11	
D12S391	20	21				
FGA	22	23				

Huh-7-STR Profiling Report

中乔新舟
CELL RESEARCH

Certificate of STR Analysis

D2S1338	19	19				
D21S11	30	30				
D18S51	15	15				
D8S1179	14	14				
D3S1358	15	15				
D6S1043	13	15				
PENTAE	11	11				
D19S433	13	14				
PENTAD	12	12				

Huh-7-STR Profiling Report

中乔新舟
CELL RESEARCH

Certificate of STR Analysis

其他说明：

(一) 分型方案及位点分布：

附表：实验方案及位点

	方案 1	方案 2	方案 3	方案 4
1	TH01	TPOX	D3S1358	AMEL
2	D12S391	VWA	D13S317	D5S818
3	D7S820	D8S1179	D6S1043	D2S1338
4	CSF1PO	PENTAD	D16S539	D21S11
5	FGA		D19S433	D18S51
6	PENTAE			

Huh-7-STR Profiling Report

中乔新舟
CELL RESEARCH

Certificate of STR Analysis

Applied Biosystems
GeneMapper 4.0

Cell Line Authentication 1213

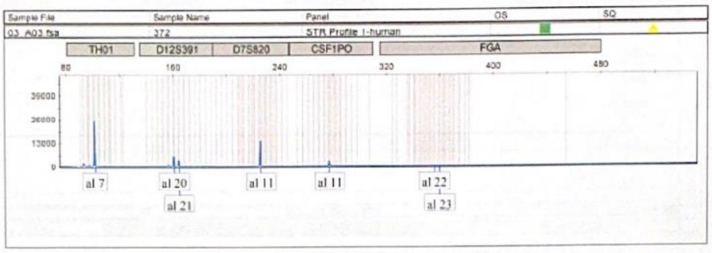

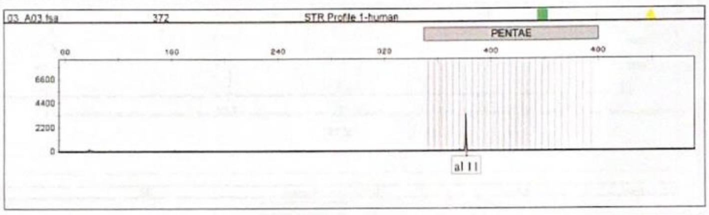

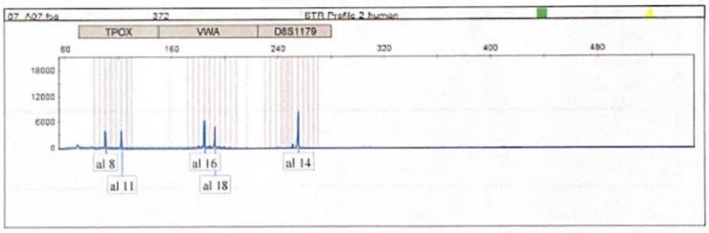

Huh-7-STR Profiling Report

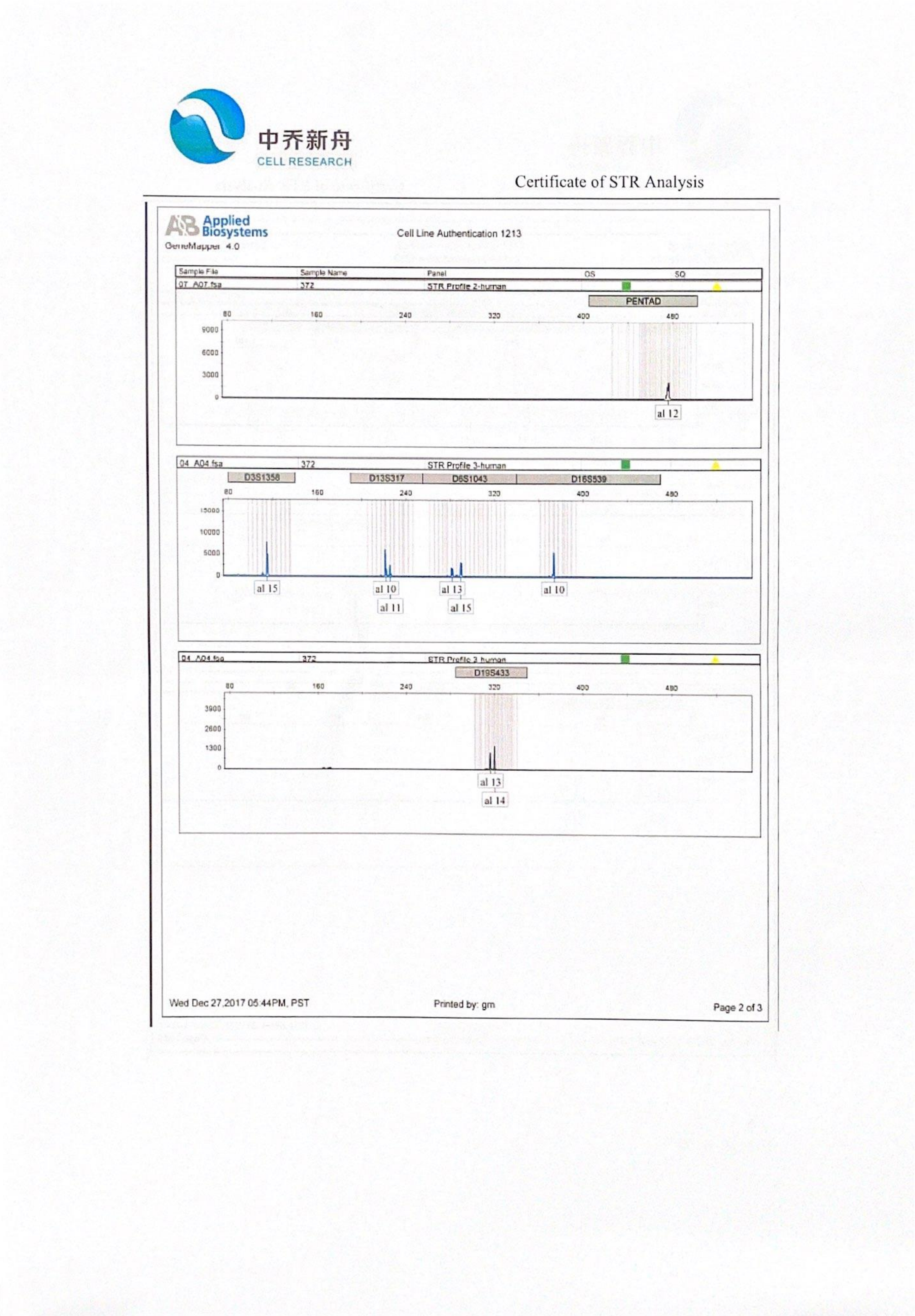

Huh-7-STR Profiling Report

中乔新舟
CELL RESEARCH

Certificate of STR Analysis

Applied Biosystems
GeneMapper 4.0

Cell Line Authentication 1213

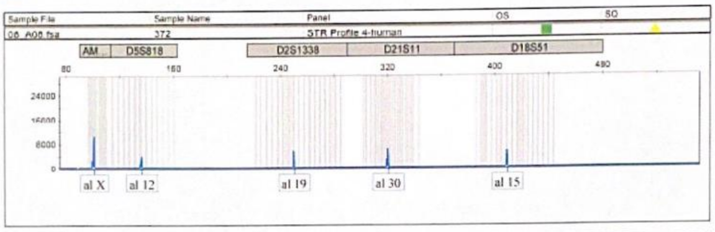

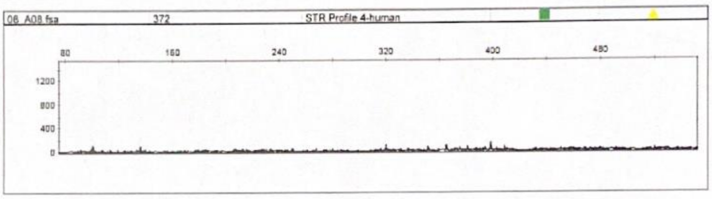

Wed Dec 27,2017 05:44PM, PST

Printed by: gm

Page 3 of 3

签发日期:
2017 年 12 月
